# Neuroinflammatory Process Involved in Different Preclinical Models of Chemotherapy-Induced Peripheral Neuropathy

**DOI:** 10.3389/fimmu.2020.626687

**Published:** 2021-02-04

**Authors:** Giulia Fumagalli, Laura Monza, Guido Cavaletti, Roberta Rigolio, Cristina Meregalli

**Affiliations:** ^1^ Experimental Neurology Unit, School of Medicine and Surgery, University of Milano-Bicocca, Monza, Italy; ^2^ NeuroMI (Milan Center for Neuroscience), University of Milano-Bicocca, Monza, Italy

**Keywords:** neuroinflammation, immune cell activation, neuropathic pain, chemotherapy-induced peripheral neuropathy, immune modulation

## Abstract

Peripheral neuropathies are characterized by nerves damage and axonal loss, and they could be classified in hereditary or acquired forms. Acquired peripheral neuropathies are associated with several causes, including toxic agent exposure, among which the antineoplastic compounds are responsible for the so called Chemotherapy-Induced Peripheral Neuropathy (CIPN). Several clinical features are related to the use of anticancer drugs which exert their action by affecting different mechanisms and structures of the peripheral nervous system: the axons (axonopathy) or the dorsal root ganglia (DRG) neurons cell body (neuronopathy/ganglionopathy). In addition, antineoplastic treatments may affect the blood brain barrier integrity, leading to cognitive impairment that may be severe and long-lasting. CIPN may affect patient quality of life leading to modification or discontinuation of the anticancer therapy. Although the mechanisms of the damage are not completely understood, several hypotheses have been proposed, among which neuroinflammation is now emerging to be relevant in CIPN pathophysiology. In this review, we consider different aspects of neuro-immune interactions in several CIPN preclinical studies which suggest a critical connection between chemotherapeutic agents and neurotoxicity. The features of the neuroinflammatory processes may be different depending on the type of drug (platinum derivatives, taxanes, vinca alkaloids and proteasome inhibitors). In particular, recent studies have demonstrated an involvement of the immune response (both innate and adaptive) and the stimulation and secretion of mediators (cytokines and chemokines) that may be responsible for the painful symptoms, whereas glial cells such as satellite and Schwann cells might contribute to the maintenance of the neuroinflammatory process in DRG and axons respectively. Moreover, neuroinflammatory components have also been shown in the spinal cord with microglia and astrocytes playing an important role in CIPN development. Taking together, better understanding of these aspects would permit the development of possible strategies in order to improve the management of CIPN.

## Introduction

Chemotherapy-induced peripheral neurotoxicity (CIPN) may occur in patients undergoing antineoplastic therapy, frequently being the most severe side-effect. CIPN is characterized by severe and long lasting symptoms that might affect daily activities and impact on patient quality of life. This clinical situation leads to drug schedule modification, or even withdrawal, thus potentially affecting patient’s survival and clinical outcome (1–4). The chemotherapy drugs act on different structures of the peripheral nervous system (PNS), due to both the reduced blood-nervous tissue barrier efficacy and the presence of fenestrated capillaries in dorsal root ganglia (DRG), targeting the axons, inducing a length-dependent axonopathy, or DRG neurons, leading to a neuronopathy (5, 6). Moreover, a large body of knowledge suggests the direct neurotoxic effect of antineoplastic agents to the central nervous system (CNS) (7).

CIPN is mainly a sensory and length-dependent neuropathy which progresses from the distal to the proximal regions (6): patients manifest paresthesia and dysesthesia, which may evolve into numbness, sensory loss, tingling, pins and needles sensation, with a stocking-and-glove distribution. Hyperalgesia or allodynia in limb extremities (neuropathic pain) may also occur (8). Rarely, a motor or an autonomic impairment is present (1, 4, 9). In most patients, CIPN becomes a chronic condition and the symptoms may persist or even progress for months after the end of the therapy, a phenomenon known as “coasting” (10).

Numerous factors are related to CIPN establishment: the type of employed chemotherapeutic agent, the administered dose, the period and schedule of treatment can contribute to the symptoms incidence and severity (11).

Although the antineoplastic mechanisms of action of neurotoxic drugs are well established, the reasons for axonal and ganglion damage remain unclear. Several neurotoxic mechanisms have been proposed including mitochondrial damage, impairment of axonal transport, oxidative stress, and involvement of drug transporters (3, 4, 12).

Recent findings suggest that neuroinflammation may have a role in CIPN. In fact, besides its action on dividing immune cells, chemotherapy treatment can lead to modulation of several immune system elements, from the cytokine expression to immune cell intracellular pathways, thus leading to neuroinflammation development and sensory nervous system sensitization (13–16).

In this context, an important role of glial cells has also been reported: satellite glial cells (SGCs) in DRG and Schwann cells in the axons are able to change their phenotype and secrete mediators, which cause neuronal excitability leading to pain hypersensitivity. The release of pro-inflammatory cytokines and chemokines may also recruit the monocytes population participating in the inflammatory response. Glial cells express the cytokine receptors, but they can also contribute to neuroinflammation with the same cytokine release, in a sort of self-sustaining system.

Moreover, the involvement of microglia and astrocytes in the neuropathic pain establishment has been demonstrated also in the central nervous system.

The features of the neuroinflammatory process are different and depend on the type of the anticancer drug (platinum derivatives, taxanes, vinca alkaloids, and proteasome inhibitors) (11, 14, 17).

Therefore, the aim of this work is to review the aspects of neuroinflammation in different classes of antineoplastic drugs. To follow this purpose, a review in the PubMed database has been carried out.

For each class of antineoplastic agents the searching entry was: ((((chemotherapy-induced-neuropathy) OR polyneuropathy) OR neurotoxicity) OR neuropathy) AND ((((((((((((((((((neuroinflammation) OR immune-cell-activation) OR immune-mediated-process) OR immune-system) OR immunomodulation) OR innate-immune-response) OR adaptive-immune-response) OR central-glial-activation) OR cytokine) OR chemokine) OR proinflammatory-mediators) OR neuroimmune-interaction) OR inflammasomes) OR glial-cell) OR macrophages) OR immune-cells) OR lymphocytes) OR neutrophils). The selected filters were used: English language and papers dated between January 2010 and May 2020. The resulting abstracts were carefully reviewed and relevant full-text manuscripts were selected. Moreover, the reference list of selected articles were searched for further relevant papers.

### Platinum Derivatives

Cisplatin (CDDP), carboplatin (CBP) and oxaliplatin (OHP) are platinum-based antineoplastic agents used as main or adjuvant treatment for solid tumors such as germ cells, lung, colorectal, gastric, breast and head and neck cancers. They are alkylating agents thus exerting their activity forming DNA-platinum adducts (12).

Even if the incidence and severity of neurotoxicity may be different among platinum compounds, it is one of their most common dose-limiting side effects (18). In fact, prolonged CDDP exposure results in the onset of a pure sensory neuropathy with a stocking-and-glove distribution characterized by the dysfunction of fine sensory-motor coordination, numbness and paresthesia. OHP infusion may result in both chronic and acute neurotoxicity. The acute neurotoxicity occurs in about 90% patients in the next hours after OHP administration and it is characterized by dysesthesias and paresthesias exacerbated by cold exposure. The OHP chronic neurotoxicity shares the same symptoms of CDDP-associated CIPN (18, 19).

Despite during the last years several neurotoxicity mechanisms have been investigated, we will point out only the evidence for different immune system elements involvement in CIPN such as leukocytes recruitment, cytokine production and signal transduction pathways.

At the early stage of OHP treatment, pain hypersensitivity has been described together with systemic immunological response characterized by an increase in circulating CD4^+^ and CD8^+^ lymphocytes, an increase in IL-4^+^ splenocytes and a decrease in regulatory T-cell (T-reg) in the inguinal lymph nodes. Except for the changes in the lymph node T-cell count, all the reported altered features returned to control values at the peak stage of pain sensitization. However, systemic depletion of T-reg cells did not exacerbate mechanical allodynia in OHP-treated mice suggesting they may not contribute to the development of neuropathic pain in OHP-induced CIPN (OIPN). Moreover, at peak stage no alteration in the cytokine serum levels was reported (20). In contrast, other authors demonstrated an increased serum or plasma levels of pro-inflammatory cytokines such as TNF-α, IL-1β and IL-6 following OHP and CDDP treatment (21–28). While Li and colleagues reported that in OIPN, T-reg reduction contributed to the onset of CIPN through the increase in the pro-inflammatory response (29), Wan and coworkers observed an increase of T-reg and a decrease of Th17 levels in OHP-treated animals (24).

Besides circulating and lymph node T-cell ratio changes during CIPN course, Laumet and colleagues demonstrated that the resolution of CDDP-induced neuropathic pain and intraepidermal nerve fibers (IENF) density reduction depended on an active endogenous process that is mediated by CD8^+^ T-cells. However, the neuroimmune mechanisms responsible for CD8^+^ T-cells mediated CIPN symptoms resolution still need to be elucidated (30). T-cells were also implicated in the prevention of CDDP-induced pain-like behavior and mitochondrial dysfunction in DRG neurons, which was exerted by histone deacetylase 6 inhibitors (31).

Besides revealing direct effects on the immune cells and cytokines production, OHP and CDDP may affect inflammatory compartment modulating gene expression. In particular, it has been reported that CDDP may affect the expression of genes implicated in neuroinflammatory processes such as TNF-α and cytokine-cytokine interactions pathways in cultured rat sensory neuron-like cells (32). In agreement with these findings, the transcriptome analysis of lumbar DRG of CDDP-treated mice revealed changes in the expression of genes involved in both neuronal damage and inflammatory processes (33). Unexpectedly, OHP treatment did not induce any alteration in the transcription of inflammation related genes suggesting that in OIPN neuronal damage might precede the inflammatory process establishment, which in turn might be responsible for the development of more chronic neuropathy symptoms (33).

Studies explored the cellular signaling pathways involved in cytokine release through the activation of the Toll Like receptors (TLRs), the innate immune system key mediators (34). TLRs are transmembrane and intracytoplasmic proteins being normally implicated in the detection of pathogens. Once activated, they induce a downstream activation of several molecules such as mitogen-activated protein kinase (MAPK), phosphoinositide 3 kinase (PI3K), nuclear factor kappa-light-chain-enhancer of activated B cells (NF-κB) and the activation transcription factor 3 (ATF3) protein. ATF3 not only regulates intracellular cascades initiated by TLRs activation, but it is also considered a marker for nerve injury. In particular, CDDP-induced tactile allodynia was associated with the upregulation in ATF3 expression in DRG neurons of CDDP-treated mice as well as in DRG neurons and sciatic nerves of OHP-treated animals (35–38). Since ATF3 plays an important role downstream TLRs activation, further studies deepened the implication of this signaling pathway in the CIPN pathogenesis. These studies on the role of TLR3 and 4 and their adapter proteins (MyD88 and TRIF) showed that CDDP-induced mechanical allodynia was reduced in *trl3* ^-/-^ and *tlr4* ^-/-^ mice compared to WT animals and was abolished in animals that completely lack TLR pathways (Myd88/Trif^lps2^ mice). Taken together, these results suggest that MyD88 and TRIF signaling cascades triggered by the activation of TLR4 and TLR3, participated in the onset of neuropathic pain induced by CDDP (39, 40).

Moreover, TLRs-pathways seem to be also involved in OIPN. In fact, OHP determined a neuroinflammatory state in DRG neurons by the increase in matrix metalloproteinase-9 (MMP-9) levels due to TLR4/PI3K/akt signaling cascade activated by high-mobility group box 1 (HMGB-1), which was released by neurons and macrophages. Furthermore, the MMP-9-dependent inflammatory process in the DRG was also associated with the activation of microglia (increase of Iba-1 immunoreactive cells) in the spinal cord indicating central sensitization (41). In contrast, other authors suggested that HMGB-1 from non-macrophage cells played a key role in the onset of OIPN, probably through the activation of TLR4, RAGE and CXCL12/CXCR4 signaling (42). MyD88 signaling pathway results in the activation of MAPK, PI3K and NF-κB, whereas TRIF dependent signaling pathway results in the production of type I interferon and a delayed activation of NF-κB. Both pathways induced an increased expression and release of pro-inflammatory cytokines and chemokines in both the central and peripheral nervous system (17, 40).

Cytokines are small molecules involved in the immune response, which are released not only by immune cells, but also by glial and neurons. They can directly or indirectly act on primary afferent fibers, DRG and spinal dorsal horn neurons leading to pain sensitization (43). In particular, a significant increase in IL-1β, IL-6, and TNF-α was reported in DRG neurons after OHP or CDDP treatment (38, 44–47). This increased expression/release of cytokines might be also associated with changes in chemokine expression.

Chemokines are a family of chemoattractant cytokines that play an important role in the activation and infiltration of macrophages and glial cells in the onset of neuropathic pain. Several chemokine contributions in platinum compounds-dependent neurotoxicity have been shown. In fact, OHP induced an increased expression of CCL2 and its receptor (CCR2) at early time points in DRG neurons, indicating that these small proteins were involved in the onset of pain caused by antineoplastic agents (48). In addition, the NF-κB p65-mediated upregulation of CX3CL1 induced an increased neuronal excitability and contributed to the development of chronic pain after repeated OHP injections (49). Furthermore, the increased expression of CXCL12, induced by IL-1β and TNF-α -mediated activation of transcription-3 (STAT3), played a critical role in the pathogenesis of OIPN (50). On the other hand, other authors reported only a significant reduction of CCL4 in DRG neurons without any additional alteration in the profile expression of cytokines and chemokines (20).

In particular, IL-8 was identified to have a relevant role in OIPN. In fact, Brandolini and colleagues reported glial activation (increase in GFAP and Iba-1 expression) and an increase in IL-8 expression associated with the activation of different intracellular signaling pathways (p-FAK, PI3K/p-cortactin, p-STAT3, COX2 and ERK1/2). These effects were attenuated by the coadministration of DF2726A, a selective inhibitor of IL-8 receptors, indicating that the increased expression of IL-8 in DRG neurons triggered a pro-inflammatory response leading to the activation of pathways implicated in microtubule stabilization, terminal axonal arborization, synaptic plasticity and cellular damage (51). Moreover, these data confirmed previous observations which suggested COX2, PI3K/Akt2, PI3K/mTOR and pERK signaling involvement in the onset of OHP-induced pain sensitization (45, 52, 53).

Besides these findings, the increased levels of pro-inflammatory cytokines, CCR2, COX2, p-ERK and p38 MAPK were associated to the decrease in protein and mRNA levels of calcineurin (CaN) and to an increase of Nuclear Factor of Activated T cells (NFAT). These findings indicated the role of the CaN/NFAT pathway in the onset of OIPN (46).

Moreover, other and specific intracellular pathways were suggested to be implicated in platinum-induced CIPN onset, such as p38 MAPK and Sphingosine 1-phosphate (S1P) receptor pathway. The incubation of SGC culture with a p38 MAPK specific inhibitor induced a decrease in PGE_2_ concentration in the medium of CDDP co-treated cells. These data suggested that the CDDP-driven p38 MAPK phosphorylation led to an increase of PGE_2_ release by glial cells that in turn stimulated the activation of PGE receptors expressed on the ganglia neurons cell membrane. Therefore, this process could modulate the neuronal activity through the activation of second messenger cascades resulting in TRPV1 activation and thus sensitization of sensory neurons (54). This hypothesis was further supported by the results of Kuai and colleagues reporting increased levels of p-p38 together with an increased expression of TRPV1 in DRG, spinal cord, trigeminal ganglion and foot skin of CDDP-treated rats showing CIPN related symptoms (28).

On the other hand, S1Ps are lipid signaling molecules that play a crucial role in different cellular processes by interacting with one of their five receptor subtypes (S1PR_1_, S1PR_2_, S1PR_3_, S1PR_4,_ and S1PR_5_). It was recently observed that the selective S1PR_2_ inhibition reduced CDDP-induced tactile allodynia and the associated activation of SGC in DRG neurons probably through the activation of stress-response proteins such as ATF3 and heme oxygenase-1 (HO-1) (55). In addition, an analogue of PGE_1_ reduced OHP-induced mechanical allodynia starting from early treatment stage (56).

In DRG neurons and peripheral nerves of OHP-treated animals, no signs of infiltrating inflammatory cells were reported claiming for the involvement of resident immune/glial cells inflammatory response with no roles for T-reg cells subset in OHP-induced mechanical allodynia (20, 36, 42). On the other hand, a huge macrophages infiltration was observed in DRG neurons of other OIPN models (29, 41). In particular, Li and colleagues reported macrophages infiltration and an increased release of IL-1β in lumbar DRG of OHP-treated mice, which was prevented by the co-administration with Bee Venom derived phospholipase A2 (bvPLA_2_). These preventive effects were reversed by the depletion of T-reg cells indicating that they were required for the anti-inflammatory response (29).

Even if DRG neurons represent the main targets of platinum neurotoxicity, SGC and Schwann cells in the PNS have been also described as relevant costars into neuroinflammation onset.

SGCs wrap the cell body of sensory neurons in the ganglia of the PNS. Following peripheral nerve injury or inflammation, SGCs are activated and release molecules that play a key role in the onset of pain conditions. In recent years, alterations in SGCs morphology and function following platinum treatment as well as their involvement in the onset of OHP-related neuropathy were evaluated. More in detail, an increased expression of GFAP-positive SGCs activated cells in DRG neurons of OHP-treated mice was reported and it was associated with an increase in gap junction-mediated coupling. Moreover, the administration of a gap junction blocker abolished the tactile allodynia and reduced the coupling percentage between SGC in OHP-treated mice. These data indicate that the increase of SGCs coupling contributed to the onset of mechanical allodynia and it is part of SGCs activation process (57). In support to these data, it has been recently reported that the incubation of SGCs primary cultures with OHP induced SGCs activation (increase of GFAP-positive cells), SGCs morphological changes, the increased expression of one of the main components of gap junctions (connexin Cx43) and pro-inflammatory cytokines release. Moreover, the incubation of DRG neurons primary cultures with the medium from OHP-treated SGCs culture enhanced neurons hyperexcitability demonstrating that these effects were due to the pro-inflammatory cytokines released by the OHP-activated SGCs (58). The activation of SGCs (increase in GFAP expression) after OHP administration was also confirmed by several other authors (36, 38, 59). In addition, it was reported that in CDDP-treated SGCs primary cultures the activation of p38 MAPK cascade enhanced the release of PGE_2_. Interestingly this event was attenuated by the application of drugs with glial modulatory activity, such as Ibudilast and SKF_86002_ (54).

With regard to peripheral nerves, the histopathological analysis of sciatic nerves of CDDP-treated rats revealed tissue damage and apoptotic alterations with axonal degeneration and myelinated fibers loss together with an increased expression of TNF-α (60). Moreover, morphological and functional alterations of Schwann cells were reported in CDDP-treated mice (61) and *in vitro* after OHP or CDDP incubation (37, 62).

Inflammatory processes were evaluated also at the level of skin hind paws. The reduction of IENF density associated with OHP regimen was first described in 2011 in the skin biopsies of hind paws, and it is often associated with the presence of hypersensitivity to a mechanical stimulus. Both effects were prevented by the treatment with minocycline (a drug with anti-inflammatory properties). These findings suggest that the epidermal denervation played an important role in the OHP-related neuropathic pain persistence and that it was caused by the release of pro-inflammatory cytokines induced by OHP (63). Since the IENF loss corresponded to an increase in IL-8 levels, the progressive accumulation of IL-8 in the epidermidis and the activation of the downstream pathways might be responsible for the OHP-associated epidermal denervation (51). Moreover, an increase in mast cells was reported in the dermis and subcutaneous tissue of plantar skin of OHP-treated mice (64, 65). In the authors’ opinion, the migration of mast cells in the skin may be caused by the increased expression of mast cell migration-related factors in keratinocytes, which were triggered by the Substance P (SP) released from capsaicin-sensitive sensory neurons (65). In CDDP-treated rats the decrease of IENF density and neuropathic pain symptoms were associated with the increased expression of TNF-α, IL-1β and PGE_2_ in paw homogenate (28).

The relationship between platinum compounds and the CNS is multi-faceted. Little information is specifically available on CDDP-induced neuroinflammation in the spinal cord whereas the most literature refers to OHP. Regarding CDDP, Park and colleagues reported no increase of Iba-1 and GFAP positive glial cells in the lumbar spinal cord of CDDP-treated animals indicating no microglial and astrocyte activation (35). In contrast to these findings, spinal microglia (increase of Iba-1 immunoreactive cells), but not astrocytes, activation associated with an increased expression of pro-inflammatory cytokines such as IL-1β, IL-6 and TNF-α, and chemokine CCL3 were also reported (66, 67). An increased expression of pro-inflammatory cytokines after CDDP treatment was confirmed also by other works (68).

With regard to OHP, no T-cell infiltration was reported in the dorsal and ventral horn of the spinal cord of OHP-treated animals (20, 69). The main interest was focused on debating about glial cells activation, inflammatory response and its cellular pathways. Therefore, it was shown that both astrocytes and microglia were activated in the early phase of OHP-related tactile and thermal hypersensitivity, whereas only astrocytes persisted in a reactive status in the late phase of OIPN (36, 37, 59, 70, 71). These data remain at least in part controversial, since some authors did not observe microglia activation at any time or demonstrated the lack of microglia activation in the late phase of OHP-induced pain sensitization (72, 73). On the other hand, other groups agreed with Di Cesare Mannelli and colleagues reporting the activation of both astrocytes and microglia (increased expression of GFAP and Iba-1 immunoreactive cells) in association with an increased release of pro-inflammatory cytokines (TNF-α and IL-1β) (38, 74). In contrast with all previous findings, Makker and colleagues did not observe neither glia (astrocytes and Iba-1 positive microglia) activation following OHP treatment, nor changes in cytokines and chemokines expression profile (20, 75). Interestingly, in the last case they showed a reduction in P2ry12 positive microglia, suggesting that homeostatic microglia reduction could result in pathological processes leading to pain hypersensitivity (20). Several studies are focused on the astrocytes activation role and the subsequent increase in pro-inflammatory cytokines (76, 77). In the spinal cord of OHP-treated rats the increase of GFAP-positive astrocytes was associated with neuropathic pain onset (72). In fact, both astrocyte activation and mechanical allodynia were reverted by the treatment with minocycline, which reverted the inflammatory response induced by activated astrocytes. Moreover, OHP-induced astrocyte activation led also to an increased expression of gap junction protein Cx43 (78). Carbenoxolone is a drug with a gap junction decoupler activity. The inhibition of astrocyte activation with carbenoxolone administration abolished the formation of astrocyte-astrocyte gap junction connections as well as the onset of allodynia in response to a mechanical stimulus. The inhibition of astrocyte-astrocyte gap junction connection with carbenoxolone pre-treatment abolished astrocytes activation as well as the onset of allodynia in response to a mechanical stimulus. In contrast, the authors did not report any protective effects when carbenoxolone was given in rats with established OIPN, suggesting that astrocyte gap junctions play a key role in the OIPN establishment, but not in its maintenance (79). Furthermore, other authors deeply elucidate the mechanism leading to pain sensitization (69, 80, 81). They observed that the astrocyte hyperactivation was accompanied by an increased release of TNF-α and IL-1β and a reduction of IL-10 and IL-4, which was not related to T-cell infiltration. Moreover, the activation of A_3_ adenosine receptor (A_3_AR) induced by the administration of a selective agonist, IB-MECA, blocked the onset of mechanical allodynia. This effect was mediated through the modulation of inflammatory processes such as inhibition of astrocyte activation and the modulation of pro-inflammatory as well as anti-inflammatory cytokines release (69). In this context, the onset of OHP-driven neuropathic pain was caused by the dysregulation of the extracellular adenosine signaling at the A_3_AR level. In fact, in a subsequent study, it was demonstrated that the dysregulation of this pathway depended on the increased expression of adenosine kinase (ADK) in astrocytes together with the increased expression of NLRP3 (NOD-, LRR- and Pyrin domain-containing protein 3). NLRP3 is an intracellular signaling molecule activated by danger signals to constitute the inflammasome. This resulted in the formation of the active form of IL-1β which in turn reduced the expression of anti-inflammatory molecules (81). Lastly, they excluded the role of GSK3β pathway in the onset of OIPN (81). Since GSK3β seems to be implicated in PTX-related CIPN, these results may indicate that different antineoplastic agents could activate different mechanisms leading to neuropathy (82, 83).

The increased level of pro-inflammatory cytokines in the spinal dorsal horn of OHP-treated rats was also correlated with the activation of the CaN/NFAT signaling (46). In addition to these findings, Huang and colleagues reported that among the different cytokines and chemokines investigated in the spinal cord [INF-γ, TNF-α, IL-1β, IL-6, monocyte chemoattractant protein-1 (MCP-1) and CX3CL1], NF-κB p65-mediated epigenetic upregulation of CX3CL1 played a key role in the central sensitization and the onset of acute pain like behavior following OHP administration (84).

Besides astrocytes activation (increase in GFAP-positive cells and protein levels) and the increased expression/release of pro-inflammatory cytokines, Wang and colleagues observed an increased expression of chemokines such as MCP-1 and monocyte inflammatory protein-1 (MIP-1α). This neuroinflammatory response, together with thermal and mechanical hypersensitivity, was repressed by the administration of melatonin. In the authors’ opinion, this effect could be mediated by the binding of melatonin to TLR on the surface of astrocytes. However, the involvement of spinal TLRs in the onset of OHP-associated neuropathic pain has not been elucidated yet and it should be considered for future studies (85).

Despite literature data indicate some controversial results, which may be attributed to the different animal models used, taken together all these findings suggest that OHP-induced neuroinflammation retrieved in the dorsal horn of the spinal cord (DHSC) (e.g. glial cell activation, increased release of pro-inflammatory cytokines and chemokines, reduced release of anti-inflammatory cytokines) might play a critical role in the onset of OHP-related neuropathic pain. In particular, it is undoubted that glial cells activation plays a pivotal role in the onset and maintenance of OIPN. However, literature data indicate some discrepancies since different effects have been observed at different time points on glial cells activity (increase in GFAP and/or Iba-1 positive cells density, increased expression and/or morphological changes) (86). Moreover, it is important to underline that glial cells are not only implicated in the pathological mechanism leading to CIPN, but also in the neuroprotective mechanisms. In fact, Di Cesare Mannelli and colleagues demonstrated that the modulation of astrocytes activity due to the nicotinic receptors *α*7 subtype (*α*7 nAChR) agonist effectively reduced OIPN (70). This effect was dependent on the TGF-β1 increase and glutamine synthetase release (87).

Interestingly, platinum compounds related neuroinflammatory events have been detected also in the upper CNS organs. Glial activation has been reported in some brain areas involved in pain signaling (36, 37, 70). Moreover, the increased levels of the pro-inflammatory cytokines TNF-α, IL-1, and IL-6 were reported in the cerebral cortex or whole brain homogenate of CDDP-treated rats (23, 88, 89). As suggested by the authors, this cytokines increase might have exacerbated the oxidative damage induced by CDDP through glutamate excitotoxicity or the upregulation of NF-κB expression and subsequent overproduction of cytokines. In fact, the increased transcription and translation of NF-κB gene was found to be associated with a reduction in the nuclear factor erythroid 2-related factor 2 (Nrf2) and HO-1 genes transcription and translation in the brain cortex and hippocampus of CDDP-treated animals (89, 90). A strong TNF-α and IL-1β increase associated with IL-10 decrease was also observed in the hippocampus of CDDP-treated rats with cognitive impairment (90, 91). Moreover, an increase of pro-inflammatory cytokines levels was reported in the midbrain periaqueductal gray of OHP-treated rats (92).

**Figure 1:**
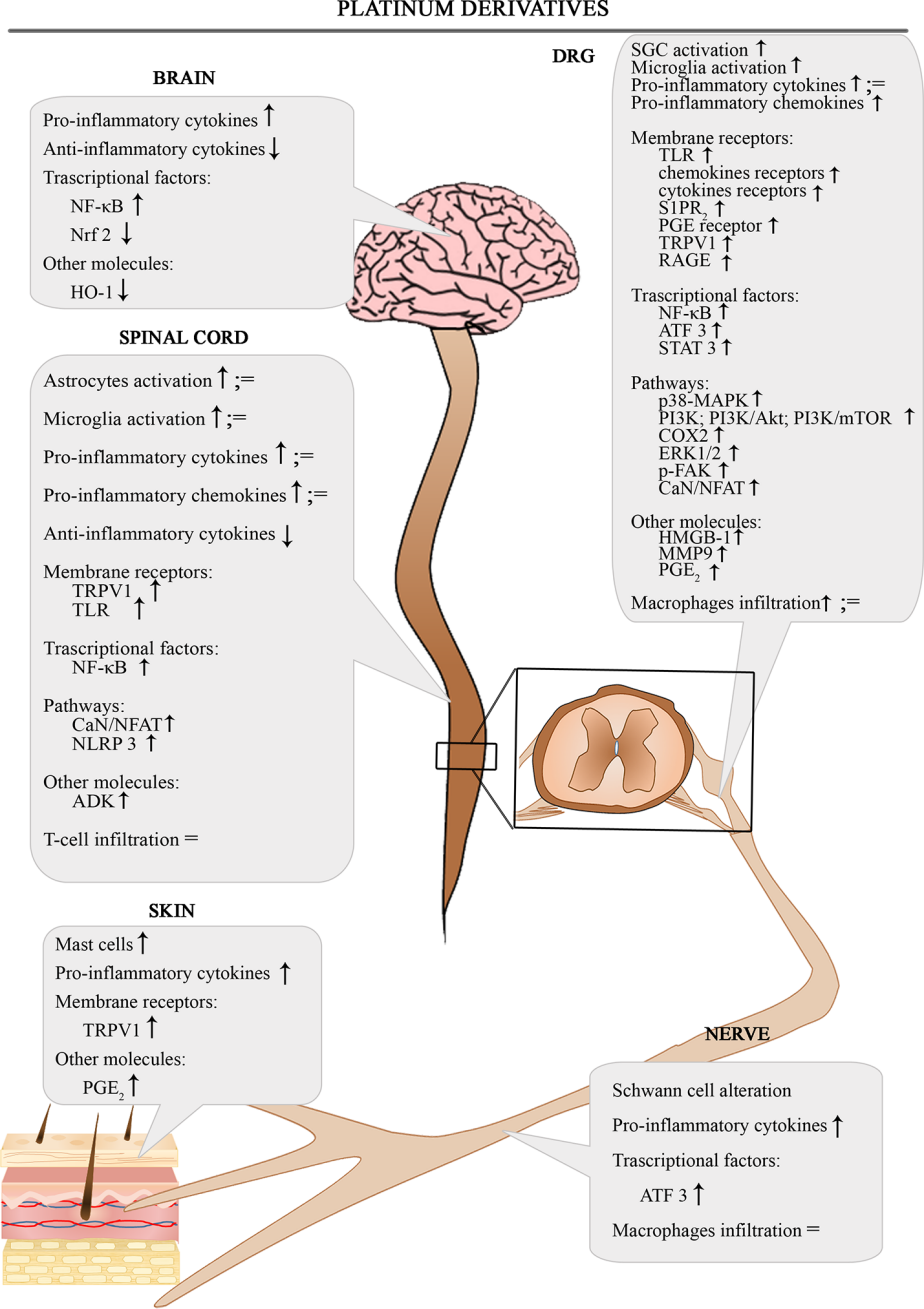
Schematic depiction of different inflammatory actors involved in the onset of platinum-induced CIPN.

The **Figure 1** shows the involvement of different inflammatory actors in the onset of platinum-induced CIPN according to the results of the most consistent studies.

### Taxanes

Paclitaxel (PTX) and Docetaxel (DCT) are the two main members of the taxane chemotherapy drug family, a class of diterpenes affecting the microtubule dynamics. Normally, microtubules undergo a process of dynamic instability, which is characterized by depolymerization and repolymerization phenomena. Taxanes exert their toxic activity binding the heterodimer β-tubulin, stabilizing the microtubules thus leading to the arrest of the cell cycle (93, 94).

PTX is an effective drug principally used as the first line choice for the treatment of breast, ovarian and lung cancer. Despite PTX is slightly less clinically effective than DCT, it is more frequently associated with CIPN (95).

The administration of PTX results in distal axonal degeneration with nerve fiber loss which results in a sensory axonal neuropathy and it is often characterized by neuropathic pain (6).

Several preclinical studies have been conducted in order to investigate the emerging concept of neuroinflammation involvement in the onset of PTX-induced CIPN (PIPN).

Most preclinical studies deal with the role of pro- and anti- inflammatory cytokines in PTX-induced pain behaviors which are mainly assessed by mechanical or thermal thresholds tests.

At present, few evidence was published about the leukocyte contribution to taxane/PTX-induced pain. As reported previously for OIPN, PTX induced a temporary increase in CD4^+^ and CD8^+^ lymphocytes only at early treatment stages (20). The specific contribution of CD8^+^ T-cells in the resolution of mechanical allodynia was demonstrated elsewhere together with their mandatory role in the up-regulation of IL-10 receptors in the DRG (96).

Pro-inflammatory cytokines and chemokines IL-1α, IL-1β, IL-6, TNF-α, INF-γ, and CCL2 were significantly increased in plasma of PTX-treated rats. The relevance of some of them (TNF-α, IL-1α, IL-1β, CCL2) in sustaining hypersensitivity or pain was demonstrated by blocking their signaling using an inhibitor or a receptor antagonist (97).

Moreover, in PTX-treated mice, IL-20 serum level was increased and this phenomenon paired with IL-20 increase in serum of cancer patients undergoing PTX therapy. IL-20 plays a pivotal role acting as inflammatory mediator in activation of monocytes and astrocytes, showing a correlation with a severe sensory pain in these patients. Targeting IL-20 signaling, using anti-IL-20 monoclonal antibody, before PTX treatment, attenuated mechanical allodynia, prevented thermal hypoesthesia and also peripheral nerve damage in PTX-treated animals. Moreover, the systemic blockade of IL-20 significantly decreased systemic inflammation suppressing the pro-inflammatory cascade activation (TNF-α, IL-1β, and MCP-1) and macrophage recruitment in DRG, correlating the role of neuroimmune system to the pain behavior. The importance of IL-20 was further confirmed by the use of IL-20R1^-/-^ mice, which were protected from PIPN and peripheral nerve degeneration (98).

Additionally, the inhibition of the IL-8 receptors (CXCR1 and CXCR2) in PTX-treated rats using a non-competitive allosteric inhibitor (reparixin) administered systemically induced a consistent antinociceptive effect (99).

Levels of pro-inflammatory elements, such as IL-6, IL-1β, TNF-α, and chemokines like CCL2, CX3CR1 were found to be modulated also in the DRG or sciatic nerves of treated animals (100–108).

Manjavachi and colleagues demonstrated an increase of the chemokine CXCL1 in both spinal cord and DRG, whereas the increase in sciatic nerve was less evident at early time points (109). In particular, Zhang and colleagues demonstrated that the blockade of MCP-1/CCR2 signaling in the DRG of PTX-treated animals attenuated mechanical hypersensitivity as well as the IENF loss (101). This observation demonstrates that mechanical hypersensitivity and IENF density reduction were dependent on the activation of MCP-1/CCR2 pathway in the DRG (101). In addition, also the treatment with minocycline prevented IENF loss as well as the development of mechanical hypersensitivity (110, 111) as previously reported for OHP. Moreover, IL-20 expression was increased in footpaw of PTX-treated animals and its inhibition prevented IENF loss caused by PTX, suggesting the role of the neuroinflammatory response in CIPN establishment also at IENF level (98).

PTX also induced the recruitment, activation and accumulation of macrophages with a pro-inflammatory M1 phenotype in DRG, leading to pro-inflammatory cytokine and chemokines release that in turn induced DRG and distal nerve ending damage (98, 112–114).

Therefore, there is a connection between neuroinflammatory elements and monocyte/macrophage infiltration: it was established that CCL2 attracts pro-inflammatory monocytes/macrophages to the DRG, causing a downstream increase of cytokines (101, 115) and M1 monocytes population triggering a pro-inflammatory cascade (98). On the other hand, despite monocyte chemoattractant CCL2 and CCL3 increased in DRG, Makker and colleagues did not observe any macrophage infiltration (20).

Distally to DRG, macrophage infiltration was also detected in sciatic nerves of PTX-treated animals (116), suggesting that macrophage activation was subsequent to the axonal degeneration (117).

Finally, it was demonstrated that PTX treatment-induced mechanical allodynia was correlated with the activation of NLRP3 inflammasome. An increased NLRP3 expression was detected in DRG and sciatic nerves of PTX-treated animals and this expression was colocalized with infiltrated macrophages (118).

Several contributions show an involvement of the CNS in the generation of the CIPN with increased levels of pro-inflammatory cytokines and chemokines in the spinal cord and the relevant role of astrocytes and microglia.

A vast number of cytokines and chemokines have been detected in the spinal cord of PTX-treated animals with main focus on IL-1β and IL-6 increase detection (20, 119, 120). In particular, the genetic interference with IL-6 signaling suggested its pivotal role in the development and maintenance of neuropathic pain (120).

Moreover, a significant increase in the expression of a robust chemoattractant molecule, CX3CL1, or of its receptor CX3CR1 (107, 121), as well as the upregulation of IL-17 expression were detected in the spinal cord of PTX-treated rats (122).

To better understand the role of cytokines and chemokines, their mRNA levels were also measured in the lumbar spinal cord of PTX-treated mice during the allodynia phase. At this later stage, an increase in the chemokine CCL2, without any concomitant change in other pro-inflammatory cytokines levels, was reported. The authors hypothesized that the alteration of those cytokines levels might have occurred at an earlier stage, contributing to the development of the neuropathy (123). An increase of TNF-α, IL-1β, IL-10, and IL-4 were instead detected in spinal cord of treated rats and associated with neuropathic pain in a study of Doyle and coworkers (124).

Among the different pathways which have been investigated in order to correlate pro-inflammatory elements and PTX-induced neuropathic pain the PKCϵ-dependent activation of TRPV1 was suggested. PKCϵ is one of the isoforms of the protein kinase C (PKC) and its activation is correlated to the augmented function of TRPV1 which enhances nociception. In particular, it was reported that mast cells release histamine which in turn induces the release of the neuropeptide SP, an inflammatory mediator that causes the sensitization of TRPV1 (125). It was demonstrated that plasma histamine levels in PTX-treated rats were higher than the control group supporting the role of histamine in PIPN. Moreover, an increased expression level of TRPV1/PKCϵ was detected in spinal cord and DRG of PTX-treated animals, hypothesizing the role of this pathway in the establishment of the animal pain behavior (126).

Besides TRPV1/PKCϵ, other studies demonstrated that the Notch signaling pathway is associated with several diseases of the nervous and immune systems. In fact, Notch signaling participates in the release of pro-inflammatory cytokines through modulation of microglial activation after nerve injury (127–130) and neuropathic pain (131). PTX treatment induced the activation of the Notch1 pathway in sciatic nerves of rats, as demonstrated by the increased expression of its receptor (106).

Moreover, IL-6 is known to activate JAK/STAT transduction pathway and MAPK cascade contributing to neuropathic pain (132). In fact, PTX treatment induced IL-6 increase in sciatic nerves, causing the increase in protein expression of its downstream molecule JAK, which in turn activated STAT3 (106, 133).

Also the activated transcription factor NF-κB was involved in inflammation, as reported in spinal cord or in sciatic nerves of PTX-treated animals. In fact, PTX caused NF-κB phosphorylation (NF-κB p65) leading to its activation and consequently to the release of pro-inflammatory cytokines that contribute to pain behavior (106, 134). More in detail, Li and colleagues suggested NF-κB p65 modulated the upregulation of CX3CL1. Interestingly, intrathecal administration of the NF-κB p65 inhibitor PDTC reduced CXCL1 expression at spinal level, as well as mechanical allodynia (121). Moreover, Akt1 (Akt family member), a downstream substrate of PI3K, once activated by CX3CR1/CX3CL1 interaction, mediates the phosphorylation of NF-κB. Akt1 levels were increased in DRG and in spinal cord after PTX treatment, playing a substantial role in painful symptoms demonstrated by the use of an intrathecal PI3K inhibitor (LY294002) which suppressed Akt1 expression and pain-related behavior (107). In addition, the inhibition of PI3K-mTOR mediated signaling led to alleviation of PTX-related neuropathic pain (102). 

Moreover, the pharmacological enhancement of SIRT1 (a histone deacetylase that regulates inflammatory responses) activation reversed NF-κB p65 phosphorylation at spinal level abolishing PTX- induced pain behavior (134).

S1P has an important role in the production of pro-inflammatory mediators such as IL-1β in the inflammation process by enhancing NLRP3 inflammasome activity (135).

In a PIPN rat model, S1PR_1_ was found to contribute to the development and maintenance of neuropathic pain activating the neuroinflammatory process through the sphingolipid pathway in the spinal cord. In particular, using a S1PR_1_ antagonist (W146), the activation of NF-κB was blocked and the release of cytokines was shifted from pro-inflammatory to anti-inflammatory phenotype (136).

A_3_AR is expressed in inflammatory cells, glial cells and neurons and it is activated by the increased production of peroxynitrite in the spinal cord (137). The activation of A_3_AR prevents the enhancement of NF-κB and MAPK pathways as well as the production of pro-inflammatory cytokines suggesting the fundamental role of spinal inflammation in PIPN (138).

As previously discussed for OIPN, the use of an A_3_AR agonist (IB-MECA) prevented neuropathic pain in PTX-treated rats by modulating spinal glial cells neuroinflammatory process (69). In fact, adenosine can modulate many biological processes, including inflammation, by activating adenosine receptors and the activation of the A_3_AR inhibits inflammatory responses in different rodent models (138). 

Other studies investigated the cannabinoid receptors in relationship with CIPN neuroinflammation. Cannabinoid receptors 1 (CB_1_) and 2 (CB_2_) belong to the endocannabinoid system and are mainly responsible for neuroinflammation suppressing effects. CB_1_ is predominantly expressed in the CNS whereas CB_2_ is mainly found in lymphoid organs and immune cells. CB_1_ has been located in several pain-related CNS regions, while CB_2_ mRNA increases after PNS damage (139).

Moreover, CB_1_ is mainly expressed in astrocytes whereas CB_2_ is involved in the activation of microglial cells (140, 141). In other studies, agonists for these receptors were employed successfully for PIPN treatment, modulating cytokines/chemokines expression and release. Several studies demonstrated that the use of CB_2_ cannabinoid receptor agonists (AM1710 and MDA7) suppressed allodynia (123, 142, 143), with a decrease of TNF-α and CCL2 mRNA levels in spinal cord of PTX-treated animals (123). In addition, glial cells activation markers and pro-inflammatory cytokines secretion were decreased in other PIPN models (143, 144) as well as a down-regulation of different pro-inflammatory coding genes was observed (145).

Moreover, the use of a synthetic cannabinoid agonist (WIN 55,212-2) to prevent PIPN development suggested the possible involvement of spinal cord glial cells in the onset of the pathology and pain modulation. Therefore, in order to study glial cell involvement in PIPN, a microglial cell activation inhibitor (minocycline) and the cannabinoid agonist were used simultaneous with the antineoplastic treatment (146). The markers of microglia cells and astrocyte activation (CD11b and GFAP, respectively) were investigated in the lumbar spinal cord of PTX-treated rats. An important astrocyte activation was detected together with the histological observations of hypertrophied cell bodies and fibrous processes, which is the typical phenotype of activated glial cells. The treatments with minocycline or cannabinoid agonist both resulted in attenuated microglia and astrocyte activation in lumbar spinal cord and in the prevention of thermal hyperalgesia and mechanical allodynia development. Furthermore, in lumbar spinal cord the IL1-β, IL-6, and TNF-α level increase was observed until the end of PTX treatment, but not later, indicating the role of these cytokines as initiators for the cascade phenomena, although at late-phase neuropathic pain and glial cell activation were maintained (123, 146).

The activation marker Iba-1 was also detected in the spinal cord of PTX-treated mice together with an increased level of IL-1β and CCL-2. In this context, the authors demonstrated the role of a selective CB_2_ receptor agonist for the prevention of Iba-1 up-regulation and for the reduction of IL-1β levels (147). Similarly, Wu and collaborators demonstrated an increase of Iba-1 expression in the dorsal horn of PTX-treated rats, confirming the role of CB_2_ receptor in the modulation of microglia dysregulation (144).

The increase of CCL2 level in spinal cord of PTX-treated mice and its contribution in hyperalgesia was also demonstrated in another study from Pevida and colleagues, in which they demonstrated a correlation with the activation of glial cells: CCL2 stimulates microglial cell activity through its receptor CCR2 (138).

A temporal correlation between microglia activation (increase of Iba-1 immunoreactive cells), the increase of chemokine CCL3 and its receptor CCR5, suggests that CCL3 release may be derived from activated microglia. Moreover, the attenuation of allodynia obtained in CCL3-neutralizing antibody-treated animals suggested that this chemokine was implicated in PTX-induced mechanical allodynia (148).

Therefore, accumulating evidence demonstrates that the activation of glial cells together with the downstream increase of pro-inflammatory cytokines and chemokines in the spinal cord is involved into the central sensitization process (20, 134, 136, 149).

Other studies demonstrated an increase of GFAP or both GFAP and Iba-1 markers in spinal cord of PTX-treated animals (124, 150–152), but controversial results have been reported. For example, other observations suggested the activation only of spinal astrocytes with no significant involvement of microglia (20, 73, 153, 154). In particular, it was suggested that the downregulation of the glial glutamate transporters GLAST and GLT-1 was responsible for the increase of GFAP-positive astrocytes (154).

The inhibition of these transporters has also been associated with neuropathic pain: in this context, the increase of GFAP expression in the spinal horn not only contributed to the mechanical and thermal allodynia in PTX-treated animals, but also to the suppressed expression of GLT-1 (82).

Finally, an increase of GFAP expression, indicating SGC activation, was demonstrated also in DRG of PTX-treated animals (57, 113).

As previously reported for OIPN, TLRs are also involved in PIPN. TLRs are differentially expressed by neurons, microglial cells and activated astrocytes (155–157).

In a PIPN rat model, TLR2 expression was downregulated and it could be involved in the antinociceptive action of the CB_2_ agonist (MDA7) in the attenuation of the pathology (145).

Moreover, in DRG neurons of PTX-treated rats the upregulation of TLR4 was demonstrated (158, 159). Its activation induced the increase of MCP-1 expression that consequently promoted macrophage infiltration into the DRG which was coincident to the development of the mechanical hypersensitivity (115). In addition, macrophages express an array of TLRs, which can stimulate the release of cytokines (155) such as TLR9, whose involvement in PIPN was at least in part sex dysmorphic (160). In fact, in male, but not female PTX-treated mice, the activation of TLR9 in macrophages results in the release of pro-inflammatory cytokines and chemokines that might activate A fibers driving CIPN mechanical allodynia. Moreover, differences were retrieved in male and female signaling downstream TLR9. In fact, male *Tlr9* mutant (deficient) animals presented a more attenuated neuropathic pain (mechanical allodynia) compared to female mice and the pharmacological inhibition of TLR9 by intraplantar or intrathecal injection of ODN 2088 reduced the pain behavior only in male (160).

**Figure 2:**
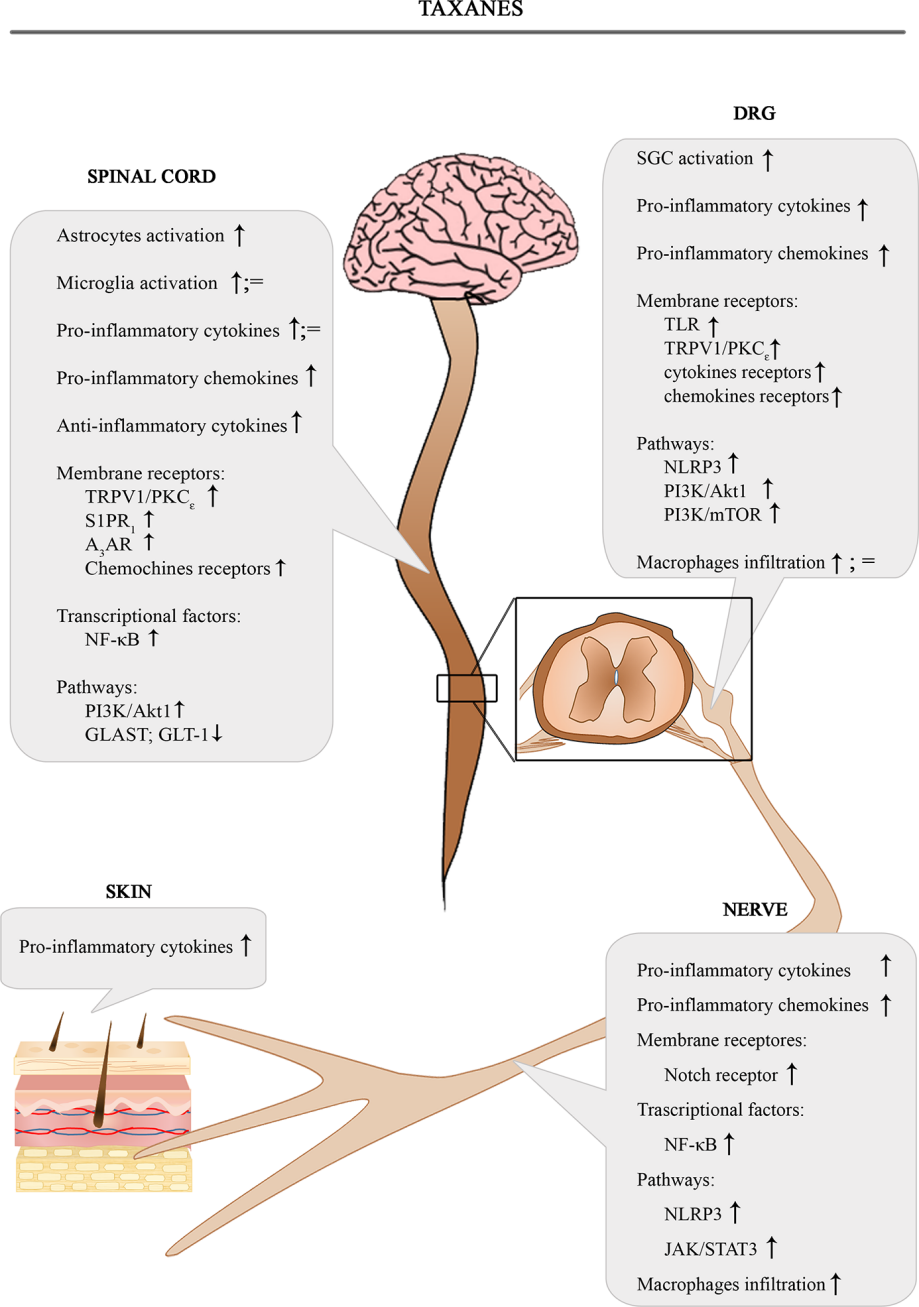
Schematic depiction of different inflammatory actors involved in the onset of PIPN.

The **Figure 2** shows the involvement of different inflammatory actors in the onset of PIPN according to the results of the most consistent studies.

### Vinca Alkaloids

Vincristine (VCR) belongs to the vinca alkaloid family and is principally used in adult and pediatric hematologic cancers such as Hodgkin’s lymphoma, non-Hodgkin’s lymphoma and leukemia (161).

VCR exerts its cytotoxic effect by binding the β-subunit of tubulin heterodimers, interfering with the microtubule formation and mitotic spindle dynamics thus leading to the arrest of dividing cells in metaphase and consequently cell death (162).

VCR elicits a strong neurotoxicity, which involves both sensory and motor fibers with also autonomic impairment. The most common side effects are numbness, paresthesia, impaired balance and weakened tendon reflexes. Regarding the autonomic dysfunctions, constipation, paralytic ileus, urinary retention and orthostatic hypotension might occur (163).

The pathogenesis of VCR-induced peripheral neurotoxicity (VIPN) is not completely understood although several studies have suggested the possible contribution of the different components of the immune system.

In several studies, the alteration of the cytokine levels in blood or PNS and CNS tissue samples correlated to VCR-related neurotoxicity. In particular, the increase of TNF-α and IL-2 levels was detected in plasma of VCR-treated rats together with the increase of TNF-α, IL-1β, IL-6 levels in sciatic nerves, in the spinal cord and brain (164–171).

Information about the anti-inflammatory cytokine IL-4 was also reported: its potential protective role was highlighted in a mice model of VIPN in which a decrease of IL-4 mRNA levels in sciatic nerves of animals together with the downregulation of p-STAT6 (the downstream effector of IL-4) were detected. This downregulation was correlated with the establishment of mechanical allodynia. The correlation was confirmed using a *IL-4* KO model in which an accelerated mechanical allodynia was evidenced and significantly decreased after IL-4 re-introduction. The re-introduction of IL-4 attenuated also p-STAT6 down-regulation suggesting IL-4 protective role towards VIPN via the stimulation of IL-4/STAT6 signaling pathway. Moreover, the over-expression of the pro-inflammatory cytokines IL-1β and TNF-α was reported in *IL-4* KO animals compared to the wild type control mice (172).

The cytokines contribution in VIPN was also reported together with monocyte/macrophage infiltration in sciatic nerves and DRG, which was evidenced by a marked increase in F4/80 or MAC-1 (173–175). Moreover, it was reported that IL-6 co-localized with invading macrophage and the use of IL-6 neutralizing antibody suppressed VCR-induced mechanical allodynia, suggesting its role in the establishment of VIPN (173).

The role of infiltrating monocytes in VIPN was also demonstrated considering the effect of VCR on both blood-nerve/brain barriers. In fact, VCR caused endothelial cells activation and tight junction disruption leading to an increase of CCR2^+^ monocytes in the spinal cord. Since microglia do not express CCR2, they were probably infiltrating monocytes. Moreover, they expressed the cysteine protease Cathepsin S (CatS) (176). It is known that monocytes release CatS which in turn solubilizes the chemokine domain of CX3CL1 and activates CX3CR1 receptor in microglia, resulting in the release of pro-inflammatory cytokines (177, 178). This cascade is involved in pain condition: in fact, the inhibition of spinal CatS reversed neuropathic pain in an animal model of sciatic nerve partial ligation (179). This last observation was afterwards confirmed in a VIPN model: a centrally-penetrant CatS inhibitor reduced VCR-induced nociception confirming that CatS acted centrally in mediating VCR action (176). 

The release of pro-inflammatory cytokines was investigated and associated with different activated pathways. An increased expression of Iba-1, CX3CR1 and P-p38 in the dorsal horn and high levels of TNF-α and IL-1β in the spinal cord were detected after VCR treatment. The chemokine CX3CL1 activates its microglia-specific receptor CX3CR1, leading to phosphorylation of p38MAPK kinase which then promotes the secretion and release of pro-inflammatory cytokines (130). In this contest, the important function of Notch signaling pathway was demonstrated in a VIPN rat model: using a Notch signaling inhibitor, the authors relieved pain behavior and downregulated the microglial pathway (Iba-1, CX3CR1 an P-p38 MAPK proteins) obtaining also a downregulation of the inflammatory factors TNF-α and IL-1β in the spinal cord (169).

The neuroinflammation role of the prokineticin (PK) family in a preclinical model of VIPN was also assessed (180). PK family is a chemokine family composed of two proteins: PK1 and PK2 with their receptors PK-R1 and PK-R2, respectively. PK-Rs are localized mostly in DRG and spinal cord where the highest density is found in the dorsal horn, suggesting their role in the central nociceptive signal transmission. PK-R1 is mostly expressed on astrocytes and microglia cells (181, 182). PK2 is an important linker element between inflammation and pain, in fact, it can modulate the immune system to a pro-inflammatory phenotype, releasing cytokines, and it can also sensitize the nociceptors (183). VCR induced an up-regulation of PK2 and PK-Rs in spinal cord and even DRG together with high levels of cytokines (IL-1β, TNF-α, and IL-6) and a significant increase of CD11b. The use of a PK-Rs antagonist (PC1) reduced the hypersensitivity within modulation of the neuroinflammation: it was able to downregulate the PK system, restoring a correct cytokine balance (180).

NF-κB-dependent CXCL1/CXCR2 signaling pathway was also shown to be relevant in VIPN (184). NF-κB is an important transcriptional factor, which regulates the release of pro- and anti- nociceptive factors, among which CXCL1. This chemokine acts through its receptor CXCR2 and has a role in central sensitization and pain maintenance (185, 186). VCR induced NF-κB activation and consequently the CXCL1 upregulation in the spinal cord of treated animals. The use of a NF-κB inhibitor led to an attenuation of CXCL1 immunostaining and a reduction of pain behavior, suggesting that NF-κB regulates CXCL1 upregulation (184). Moreover, p65 phosphorylation, and its increased expression, activated NF-κB pathway. The expression of p-p65 was increased in VCR-treated animals at spinal level together with the up-regulation of TNF-α and the down-regulation of IL-10, confirming NF-κB role in the neuro-immune modulation (164, 187).

In spinal cord, similarly to other drugs, microglia cells and astrocytes were involved and activated also after VCR treatment, with the release of pro-inflammatory elements. In this context, exogenous induction of HO-1 was suggested as a potential therapy approach. HO-1 is a rate-limiting enzyme of heme degradation whose induction protects against cytotoxicity and has a role in immunomodulatory and anti-inflammatory processes (188). HO-1 inducer attenuated VCR-induced pain hypersensitivity as well as it reduced GFAP and Iba-1 expression. Moreover, the HO-1 induction decreased the activation of MAPKs, which mediated the production of pro-inflammatory elements, such as TNF-α and MCP-1 (189).

The glial markers Iba-1 and GFAP colocalized with TNF-α demonstrating that TNF-α was released by these activated spinal glial cells and that it was at least in part responsible for VCR-induced mechanical allodynia, since the use of a neutralizing antibody against TNF-α reduced VIPN (190). On the contrary, neither microglia hypertrophy nor increase of Iba-1 levels were detected in the study of other authors (73).

As suggested also for the other antineoplastic agents, TLR-4 seemed to be implicated in VIPN. In fact, using a *Tlr4* KO mouse model, mechanical allodynia was decreased as well as in naive minocycline-treated animals (191).

**Figure 3:**
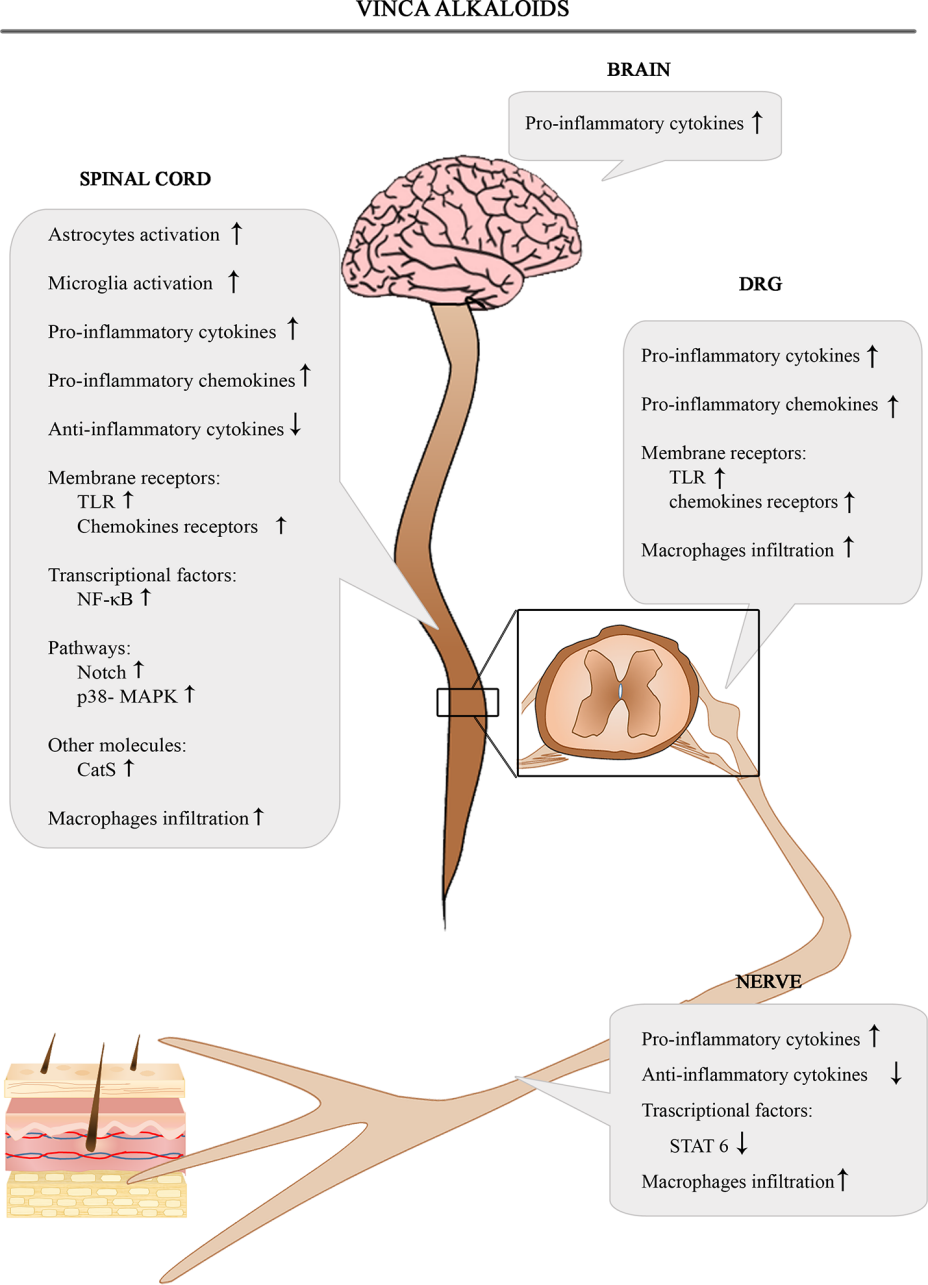
Schematic depiction of different inflammatory actors involved in the onset of VIPN.

The **Figure 3** shows the involvement of different inflammatory actors in the onset of VIPN according to the results of the most consistent studies.

### Proteasome Inhibitors

Bortezomib (BTZ) is a functional proteasome inhibitor commonly used as the frontline anticancer drug in the treatment of multiple myeloma (MM). Although its clinical effectiveness has been clearly demonstrated, it frequently leads to dose limiting painful peripheral neuropathy.

Neuropathic pain associated with BTZ is often severely debilitating, including spontaneous pain as well as allodynia and hyperalgesia in the distal extremities of limbs. Unfortunately, this devastating complication often requires BTZ dose modification or discontinuation (192, 193), compromising the clinical outcome of MM patients.

The pathophysiological mechanisms by which BTZ leads to BTZ-induced peripheral neurotoxicity (BIPN) remains largely unclear and the molecular mediators of the neuropathic pain-syndrome have not been fully elucidated.

A large body of data indicates that BTZ is involved in the development of peripheral damage related to immune-mediated processes in addition to its toxic effects (194, 195). These data focus on the pivotal role of inflammation and immune response in the development and maintenance of BIPN and some of them reported also that BIPN responded to high dose steroids or intravenous immunoglobulins (IVIg) (196, 197). All together these findings provide a rationale to identify neuroinflammation as a critical key player involved in BIPN, which includes a primary sensitization process at the level of sensory neurons in DRG up to the involvement of the spinal cord (198).

In order to study neuropathic pain-syndrome, several CIPN animal models mimicking clinical features of BIPN have been reported. As previously done for the other chemotherapeutic drugs, the inflammatory immune and immune-like glial cells as well as the actions of the pro- and anti-inflammatory mediators (i.e. nerve growth factor, cytokines/chemokines or microRNA) will be considered. In this section, we will also report some attempts that were performed in order to reduce the generation of BIPN by targeting immune and glial cell responses as well as released cytokines and chemokines.

Considering first the effect of BTZ on circulating white blood cells, no changes in cell subsets and functions have been associated with BIPN, whereas the main BTZ action has been referred to PNS. Moreover, regarding cytokine circulating molecules, BTZ injection caused increased pro-inflammatory cytokines TNF-α IL-1β, IL-6 levels in plasma (199). As previously observed in the PIPN model (115), one of the initial immune responses is mediated by resident macrophages and by a large influx of infiltrated macrophages, promoting neuropathy progression (156). Of interest, M1 pro-inflammatory phenotype macrophages in the peripheral nerves have been found to play an important role in the pathogenesis of BIPN (197). These data suggest a crucial role of macrophage into neuropathic pain maintenance, which was further supported by the depletion of macrophage infiltration due to the administration of a high dose of human IVIg able to block BTZ-induced allodynia and hyperalgesia (197).

Peripheral glial cells support an active role in immune and sensory transmission by maintaining metabolic and ionic homeostasis into the peripheral nervous system (200). They were also demonstrated to play a relevant role even in BIPN by undergoing notable phenotypic changes associated with pain hypersensitivity (201). Indeed, the glial cells activation was characterized by hypertrophy and the presence of large cytoplasmic vacuoles due to mitochondrial damage and endoplasmic reticulum enlargement in both SGCs and Schwann cells in rats undergoing BTZ treatment (201).

More interestingly, the production of pro-inflammatory mediators was positively correlated to the nociceptor sensitization and neuropathic pain-syndrome derived from changes in DRG, enhancing neuronal excitability and generating pain hypersensitivity. It is noteworthy that highest and early increase of IL-6 and TNF-α in DRG of treated mice was followed with a later increase of TGF-β1 and IL-1β, which are accompanied by elevated TNF-α receptor1 (TNFR1) induction in BIPN (202). The treatment with TNF-α neutralizing antibody, significantly prevented the BTZ-induced electrophysiological alterations and the loss of myelinated and unmyelinated fibers. Furthermore, it elicited an improvement in pain behavior which was correlated to decreased expression of TNFR1, IL-6 and IL-6- corresponding signal transducing receptor (IL-6Rα) in DRG (202).

Likewise, the dose-related effects of anti-TNF-α therapy on neurotoxicity were also demonstrated in the BIPN rat model, in which the co-administration of an antibody against TNF-α was able to revert the neuropathic symptoms, although the development of the neuropathy was not prevented (203). Accordingly, a progressive increased serum level of TNF-α in patients suffering from peripheral neuropathy after several cycles of BTZ-therapy was reported. In the same study, they also demonstrated a potential neuroprotective effect of the co-administration of anti-TNF-α treatment in the BIPN rat model, showing an improvement of both electrophysiological parameters and mechanical allodynia (204). Moreover, the authors hypothesized that increased TNF-α levels caused the upregulation of heparanase (HSPE) expression, an endoglycosidase involved in the production of inflammatory cytokines, which was secondary to the development of the neuropathy (204).

In support of the crucial role of TNF-α modulation in peripheral sensitization processes involved in BTZ-induced allodynia, Zhang and colleagues demonstrated that the increase of TNF-α expression in rat DRG paired with that of the phosphorylated JNK1/2. Indeed, the suppression of TNF-α signaling induced by the TNF-synthesis inhibitor, thalidomide, as well as by *TNFR1* and *TNFR2* depletion in KO male mice, blocked JNK1/2 activation in DRG which was accompanied by a reduction in mechanical allodynia (205). Overall, the upregulation of TNF-α appeared to play a relevant role in orchestrating activation of JNK signaling by TNFR1 and TNFR2, mediating mechanical allodynia occurrence (205).

An ongoing interaction between transient receptor potential ankyrin 1 (TRPA1), TNF-α and its receptor TNFR1 were recently demonstrated in relationship with neuropathic pain onset. Increased TRPA1 expression in rat DRG was associated with development of mechanical pain and cold sensitivity following BTZ treatment (206). Interestingly, suppressing the expression of TRPA1 by TRPA1 antagonist HC030031 reduced allodynia and thermal hyperalgesia in BTZ-treated rats. Moreover, blocking TNF-α pathway by the action of pentoxifylline resulted in attenuated p38-MAPK and JNK signal in DRG, as well as a reduction of TRPA1 expression which was correlated with a block of neuropathic pain onset (206).

Similarly, inhibition of TNFR1-TRPA1 pathway in the dorsal horn has recently been reported, including the critical involvement of the microRNA (miR-155) in painful BIPN (207). In particular, inhibiting miR-155 signal in BTZ-treated rats displayed significantly reduced mechanical and cold sensitivity associated with the decreased TNFR1 and TRPA1 expression as well as the reduction of signals p38-MAPK and JNK in DHSC. Interestingly, administration of miR-155 mimic interfered with TNFR1-TRPA1 signal and contributed to enhanced cold hypersensitivity and allodynia (207). Therefore, interfering with this complex pathway may provide an effective treatment of neuropathic pain in BTZ-treated patients.

Moreover, in a recent study by Liu and collaborators, increased TRPA1 and IL-6 receptor levels were observed in the DRG after BTZ administration. Their contribution to BTZ-induced mechanical and cold hypersensitivity was supported inhibiting TRPA1 function, as well as blocking IL-6-mediated signal transduction. This resulted in a downregulation of intracellular signal mediated by p38-MAPK and JNK in the sensory neurons, which are correlated with a decreased TRPA1 protein expression and a consequently absence of mechanical pain and cold sensitivity. Together these results suggested that IL-6 is a signal activating TRPA1 that could be a relevant key process engaged in painful BIPN, and that interfering with this pathway might be a useful tool to reduce neuropathic pain in multiple myeloma patients (208).

To better investigate their pivotal role and responsibility in the development and maintenance of painful BIPN the chemokine family and neuroinflammation pathways were thoroughly investigated. The upregulation of chemokine CCL2 expression in DRG neurons, but not in SGCs was observed in BTZ injected rats, and it was associated with a huge infiltration of macrophage, as well as an enhanced expression of the transcription factor c-Jun. This effect might be associated with co-localization of c-Jun and ATF3 transcription factors. Furthermore, blocking c-Jun signaling prevented mechanical allodynia, as well as CCL2 upregulation. More interestingly, the pre-treatment with ATF3 siRNA suppressed the c-Jun binding to the *ccl2* promoter (209).

More recently, the increasing evidence has emphasized that PKs may contribute to pain hypersensibility and neuroinflammation in a BTZ-treated mice model, as previously described in VIPN model (180). Of note, overexpression of PK system (PK2 and PK-R) in all the tissues involved in pain transmission has been proposed to contribute to the thermal hyperalgesia, mechanical and cold allodynia by inducing structural damage to DRG neurons and SGCs. Furthermore, increased macrophage activation markers and TLR4 mRNA were detected in both DRG and sciatic nerves. The increased release of pro-inflammatory cytokines and the decrease of anti-inflammatory IL-10 expression were also reported in CNS and PNS (198). In addition, the activation of the spinal cord glial cells by upregulation of glial markers GFAP led to pain hypersensibility. Moreover, DRG structural alteration together with the development of altered behavioral parameters were totally abrogated by subcutaneous administration of a PK-R antagonist (PC1) in treated mice. An evident attenuation of macrophage recruitment and prevented central sensitization into DHSC were also reported (198). Finally, the authors speculated that the upregulation of PK2 protein could be regulated by the binding between phosphorylated STAT3 and the PK2 promoter (198).

Recently, increasing attention is being paid to the role of NLRP3 inflammasome complexes as key mediators of inflammatory mechanisms involved in neuropathic pain. Accumulating evidence indicates that NLRP3 is involved in several CNS diseases, as well as in PIPN and OIPN (81, 118). In a rat model of BIPN, Liu and coworkers demonstrated an increased NLRP3 inflammasome mRNA and protein expression in DRG which could lead to painful neuropathy. This event was correlated with the upregulation of phosphorylated STAT3 signaling via increasing histone acetylation, as well as with the enhanced binding of STAT3 to *Nlrp3* promoter in DRG. To further support the importance of NLRP3 in BIPN, the intrathecal injection of NLRP3 siRNA attenuated mechanical allodynia caused by BTZ. Meanwhile, specific inhibition of STAT3 activity resulted in a suppressed upregulation of NLRP3 in DRG, thus ameliorating mechanical allodynia induced by BTZ (210).

Since there is extensive evidence supporting the role of neuroinflammation in the CNS, several immune-like glial cells changes in the spinal cord have been implicated in CIPN, identifying spinal glial cells as key players that drive the establishment and maintenance of neuropathic pain (211). For instance, in animal models of PIPN, OIPN and BIPN, astrocytes became activated and proliferated (72, 154). Furthermore, since astrocytes represent the largest CNS cell population, their morphological activation in CIPN models has been debated (72). Of note, in OIPN and BIPN models, the spinal astrocytes activation occurred at multiple time points after chemotherapy treatment in parallel with the induction of mechanical sensitivity. Moreover, an immunohistochemical study which compared the activation of astrocytes and microglia in the DHSC demonstrated that only the increase in GFAP positive astrocytes was correlated to the induction of allodynia in BTZ-treated animals. In particular, changes in spinal glial morphology (arborization and hypertrophy of astrocytes) were reported, while no activation of microglia was observed. The application of minocycline, similarly to the previously reported CIPN models (63, 79, 111), totally prevented the painful symptoms and it counteracted the astrocyte activation which may result from a common underlying mechanism (72).

A more recent research from Robinson and colleagues described significant alterations in astrocytic connexins and glutamate transporters mediated neuropathic pain in the BIPN model. As previously mentioned in various forms of CIPN (79, 154), increased GFAP-positive astrocytes is believed to be one of the critical proteins correlated with the upregulation of Cx43 and the glutamate transporter dysfunction (212). These activated astrocytes (characterized also by hypertrophy and increased arborization) displayed an initial upsurge of intracellular calcium, coinciding with the presence of synaptic glutamate due to the downregulation of glutamate transporters (GLAST). In addition, after the follow up period, an increase of phosphorylated Cx43 correlated with mechanical hypersensitivity. The administration of minocycline inhibited GFAP, GLAST, and Cx43 increase and prevented the onset of mechanical allodynia (212).

Interestingly, Guo and collaborators focused on the activation of microglia and SGCs (increase of Iba-1 and GFAP immunoreactive cells) after BTZ injection, indicating the crucial association between the purinergic ligand gated ion channel 7 receptor (P2X7R) and p38 MAPK pathway as a prerequisite for BIPN (213). P2X7R is richly expressed in glial cells. The increase of P2X7R protein levels and p38 MAPK phosphorylation were reported in DRG already 2 days after chemotherapy treatment. In particular, the authors observed that P2X7R was colocalized with GFAP in DRG and with Iba-1 in DHSC, while p38 MAPK was mainly expressed in microglia cells. P2X7R downstream pathway inhibition could be able to revert neuropathic pain and the inhibition of p38 MAPK phosphorylation led to a downregulation of P2X7R expression level in BIPN-affected animals. These results indicated that blocking P2X7R-p38 signal with pharmacology therapy was beneficial to alleviate neuropathic pain resulting from BTZ treatment (213).

Moreover, the production of various pro-inflammatory cytokines as well as chemokines by spinal cord astrocytes, caused the increase of the activity of spinal cord nociceptive neurons after BTZ treatment in both mice and rat models (192, 214). Recently, the upregulation of protein expression of TNF-α as well as its mRNA level were found in rat spinal cord neurons together with increased IL-1β expression and JNK activation in astrocytes in BTZ-induced allodynia model (215). Within the spinal cord, the predominant increased JNK phosphorylation was not correlated with a similar activation of the ERK and p38-MAPK pathways. Similarly to the work of Zhang and colleagues (205), intrathecal injection of thalidomide or the IL-1 receptor antagonist (IL-1ra) in BIPN model ameliorated mechanical allodynia by downregulation the phosphorylation of JNK signal (215).

Finally, compelling researches have indicated that altered sphingolipid metabolism may be correlated with CIPN pathology and neuroinflammation in both human and animal models (199). In particular, a clinical study showed a correlation between PIPN and plasma levels of neurotoxic sphingolipids (216), and up-regulated sphingolipid metabolism were reported in PNS in a docetaxel neurotoxicity mice model (217). In addition, there was a striking upsurge in sphingosine-1-phosphate (S1P) signaling, its receptor 1 (S1PR_1_) and dihydro-S1P in the DHSC following BTZ treatment. The development of mechano-hypersensitivity was associated with an increased TNF-α and IL-1β and an enhancement of presynaptic glutamate release in the DHSC in mice model (218). Similarly to PTX studies (69), neuropathic pain behavior induced by BTZ was attenuated by S1PR_1_ antagonists (FTY720 or NIBR14 treatments) or siRNA to knockdown the expression of S1PR_1_. In addition, the administration of BTZ in mice with astrocyte-specific deletion of S1PR_1_ did not engage neuropathic pain associated with BIPN. These data suggest that astrocyte activation by S1PR_1_-dependent neuroinflammatory signaling is a key cellular site for S1PR_1_ activity. Therefore, consistent with this concept, emerging evidence proposed that S1PR_1_ signaling pathway in astrocytes and subsequent mechanical allodynia were regulated by the activation of NLRP3 inflammasome and IL-1 signaling in DHSC (219). Otherwise, Alè and colleagues regarding the paper of Stockstill and collaborators (218) evidenced several points to consider about the model involved in this article. In particular, the authors suggested a better understanding and attention toward the cumulative dose and the animal model employed since this acute model is not representative of the chronical painful BIPN. Moreover, blinder conditions should be employed, as well as a full investigation of the complexity of PNS (220).

**Figure 4:**
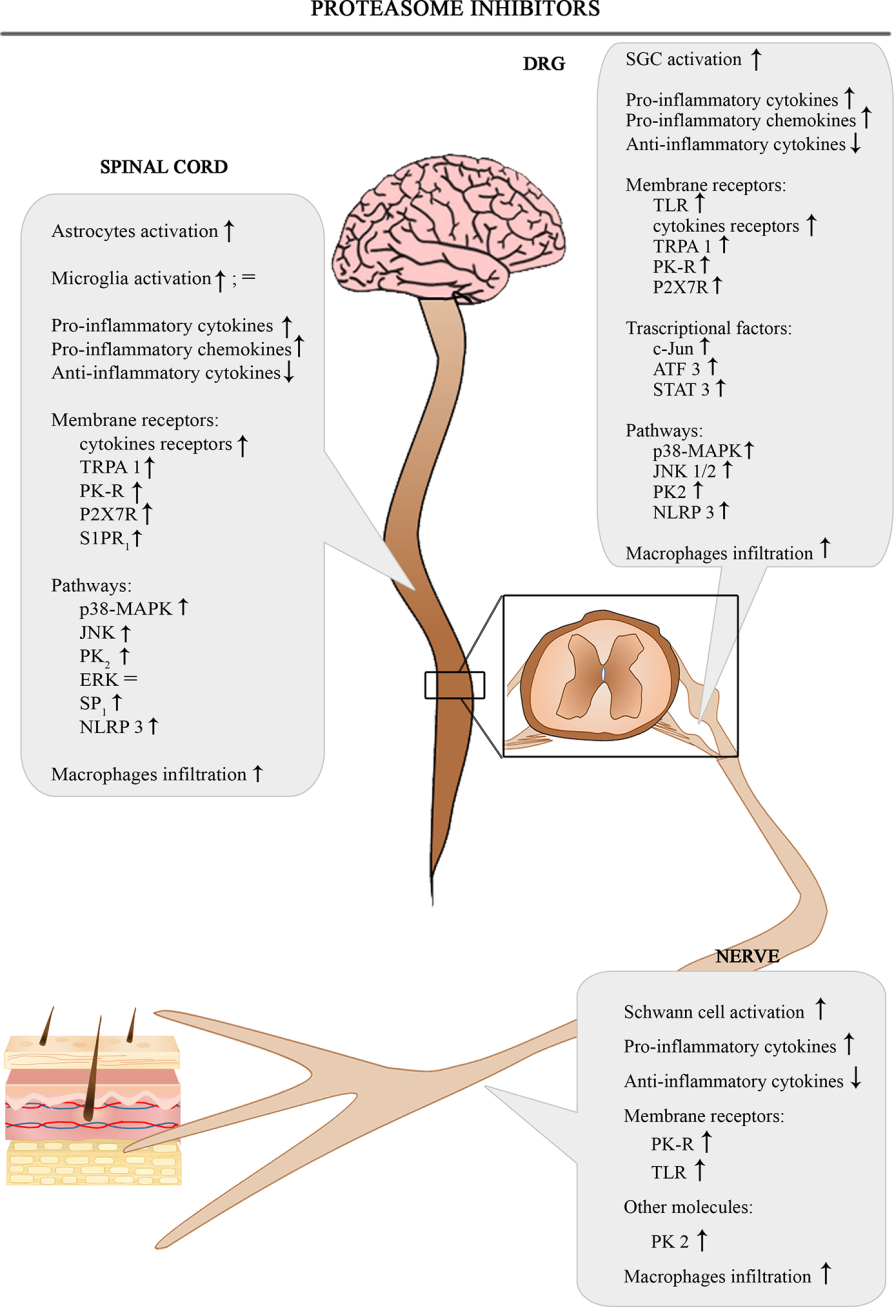
Schematic depiction of different inflammatory actors involved in the onset of BIPN.

The **Figure 4** shows the involvement of different inflammatory actors in the onset of BIPN according to the results of the most consistent studies.

## Discussion

Chemotherapy represents the only available approach for fighting many different cancer types. However, this regimen is often associated with CIPN onset that frequently results in premature interruption of the treatment. Despite the recent development of some symptoms-related therapies for CIPN, no preventive or curative interventions are available. The mechanisms underlying CIPN are complex and not fully elucidated.

Here, we focused our attention on reviewing studies which pointed out the role of neuroinflammation in the onset and persistence of CIPN in rodent models. Since the generation of pain hypersensitivity may represent a common feature in the patient undergoing chemotherapy, a crosstalk between neuro-immune balance and pro-inflammatory mediators, as well as glial activation, was considered.

Despite some discrepancy in activated neuroinflammatory pathways within the same chemotherapy agent, presumably due to the different animal models used, CIPN exhibits a peculiar immune response and phenotypic cells changes in both CNS and PNS. The reliability of preclinical models is a very debated topic in CIPN, since they could negatively impact on the translatability of the results from bench to the clinical setting. The different reported effects may depend on several aspects: the species, the strain, the age, the sex, the dosage, and the treatment schedule. In particular, high doses of chemotherapy might be useful to obtain evident histopathological alterations and increased inflammatory response, but they could be far from a clinically relevant dose (221). Indeed, several CIPN-inducer agents seem to affect the same immunological targets despite the underlying pathways may be different. The identification of the peculiar neuroinflammatory pathway for each chemotherapeutic drug is required in order to be a further weapon to fight the single drug induced CIPN. Moreover, an early detection of inflammation targets in patients' blood may serve as a promising biomarker for predicting CIPN onset and course.

As we described, the inhibition of neuroinflammation pathways by immune modulation therapy in CIPN may potentially result in clinical benefits in terms of preventing and improving the painful peripheral neuropathy related to chemotherapeutic agents.

## Author Contributions

CM and GF designed the review. GF and LM defined the search strategies and reviewed the literature. GF drafted the *Introduction*, the *Taxanes*, and the *Vinca Alkaloids* sections. LM drafted the *Platinum Derivatives* and *Discussion* sections. CM drafted the *Proteasome Inhibitors* and *Discussion* sections. GC coordinated and RR edited the manuscript. All authors contributed to the article and approved the submitted version.

## Funding

This work has been supported by Fondazione Cariplo, grants n° 2019-1482, PRIN n° 2017ZFJCS3, and IMMUN-HUB n° 1165235.

## Conflict of Interest

The authors declare that the research was conducted in the absence of any commercial or financial relationships that could be construed as a potential conflict of interest.

## Glossary

**Table:**

A_3_AR	A_3_ adenosine receptor
ADK	adenosine kinase
AKT	protein-chinasi B
ATF3	activation transcription factor 3
BIPN	bortezomib-induced peripheral neurotoxicity
BTZ	bortezomib
bvPLA_2_	bee venom derived phospholipase A2
CaN	calcineurin
CB_1_	cannabinoid receptor 1
CB_2_	cannabinoid receptor 2
CBP	carboplatin
CCL2	C-C motif chemokine ligand 2
CCL3	C-C motif chemokine ligand 3
CCL4	C-C motif chemokine ligand 4
CCR2	C-C motif chemokine receptor 2
CDDP	cisplatin
CD4^+^ T-cell	linfociti T helper
CD8^+^ T-cell	cytotoxic T-cell
CD11b	cluster of differentiation molecule 11B
CIPN	chemotherapy-induced peripheral neurotoxicity
c-Jun	Proto-Oncogene C-Jun
CNS	central nervous system
COX2	cyclooxygenase-2
CX3CL1	C-X3-C motif chemokine ligand 1
CX3CR1	C-X3-C motif chemokine receptor 1 (fractalkine)
CXCL1	C-X-C motif chemokine ligand 1
CXCL12	C-X-C motif chemokine ligand 12
CXCR1	C-X-C motif chemokine receptor 1
CXCR2	C-X-C motif chemokine receptor 2
CXCR4	C-X-C motif chemokine receptor 4
Cx43	gap junction connexin Cx43
DCT	docetaxel
DHSC	dorsal horn spinal cord
DRG	dorsal root ganglia
ERK	extracellular signal-regulated kinase
FAK	focal adhesion kinase
GFAP	glial activation marker
GLAST	glutamate-aspartate transporter
GLT-1	glutamate transporter-1
GSK3β	glycogen synthase kinase-3 beta
HMGB-1	high-mobility group box 1
HO-1	heme oxygenase-1
HSPE	heparanase
Iba-1	Ionized calcium binding adaptor molecule 1
IENF	intra-epidermal nerve fibers
IL-1α	interleukin-1α
IL-1β	interleukin-1β
IL-1Ra	interleukin-1 receptor antagonist
IL-2	interleukin-2
IL-4	interleukin-4
IL-6	interleukin-6
IL-6R	interleukin-6 receptor
IL-8	interleukin-8
IL-10	interleukin-10
Il-17	interleukin-17
IL-20	interleukin-20
IL-20R	interleukin-20 receptor
INF-γ	Interferon gammaMAPK: mitogen-activated protein kinase
IVIg	Intravenous Immunoglobulin
JAK	Janus kinase
JNK	c-Jun N-terminal kinase
MAPK	mitogen-activated protein kinase
MCP-1	monocyte chemoattractant protein-1
MIP-1α	macrophage inflammatory protein-1 alpha
MM	multiple myeloma
MMP-9	matrix metalloproteinase-9
mTOR	mammalian target of rapamycin
MyD88	myeloid differentiation primary response 88
NFAT	nuclear factor of activated T-cells
NF-κB	nuclear factor kappa-light-chain-enhancer of activated B cells
NLRP3	NOD-, LRR- and Pyrin domain-containing protein 3
Nrf2	nuclear factor erythroid 2–related factor 2
OHP	oxaliplatin
OIPN	oxaliplatin-induced peripheral neurotoxicity
P2ry12	Purinergic Receptor P2Y, G-Protein Coupled, 12
P2X7R	Purinergic Receptor P2X, Ligand Gated Ion Channel, 7
PGE_2_	prostaglandin E2
PI3K	phosphoinositide 3 kinase
PIPN	paclitaxel-induced peripheral neurotoxicity
PK	prokineticin family
PKC	protein kinase C
PKCϵ	protein kinase C-epsilon
PK-R	prokineticin family receptor
PNS	peripheral nervous system
PTX	paclitaxel
RAGE	receptor for advanced glycation end products
S1P	sphingosine-1-phosphate
S1PR	sphingosine-1-phosphate receptors
S1PR_1_	sphingosine-1-phosphate receptors 1
S1PR_2_	sphingosine-1-phosphate receptors 2
SGCs	satellite glial cells
SP	Substance P
STAT3	activation of transcription-3
STAT6	activation of transcription-6
TGF-β1	transforming growth factor beta-1
TLR	toll like receptor
TNFR1	TNF-α receptor1
TNF-α	tumor necrosis factor-alpha
T-reg	regulatory T cell
TRIF	Toll/IL‐1R domain‐containing adaptor‐inducing interferon-β
TRPA1	transient receptor potential ankyrin 1
TRPV1	transient receptor potential vanilloid 1
VCR	vincristine
VIPN	vincristine-induced peripheral neurotoxicity.
